# The Neuroimmune Interplay in Joint Pain: The Role of Macrophages

**DOI:** 10.3389/fimmu.2022.812962

**Published:** 2022-03-10

**Authors:** Daniela P. Vasconcelos, Clive Jabangwe, Meriem Lamghari, Cecília J. Alves

**Affiliations:** ^1^Instituto de Investigação e Inovação em Saúde da Universidade do Porto- Associação, Porto, Portugal; ^2^Instituto de Engenharia Biomédica, Universidade do Porto, Porto, Portugal; ^3^Faculdade de Engenharia, Universidade do Porto, Porto, Portugal; ^4^Instituto Ciências Biomédicas Abel Salazar, Universidade de Porto, Porto, Portugal

**Keywords:** inflammation, sensory innervation, sympathetic innervation, rheumatoid arthritis, osteoarthritis, aseptic loosening, macrophages

## Abstract

Chronic pain associated with joint disorders, such as rheumatoid arthritis (RA), osteoarthritis (OA) and implant aseptic loosening (AL), is a highly debilitating symptom that impacts mobility and quality of life in affected patients. The neuroimmune crosstalk has been demonstrated to play a critical role in the onset and establishment of chronic pain conditions. Immune cells release cytokines and immune mediators that can activate and sensitize nociceptors evoking pain, through interaction with receptors in the sensory nerve terminals. On the other hand, sensory and sympathetic nerve fibers release neurotransmitters that bind to their specific receptor expressed on surface of immune cells, initiating an immunomodulatory role. Macrophages have been shown to be key players in the neuroimmune crosstalk. Moreover, macrophages constitute the dominant immune cell population in RA, OA and AL. Importantly, the targeting of macrophages can result in anti-nociceptive effects in chronic pain conditions. Therefore, the aim of this review is to discuss the nature and impact of the interaction between the inflammatory response and nerve fibers in these joint disorders regarding the genesis and maintenance of pain. The role of macrophages is highlighted. The alteration in the joint innervation pattern and the inflammatory response are also described. Additionally, the immunomodulatory role of sensory and sympathetic neurotransmitters is revised.

## 1 Introduction

Joint diseases, such as osteoarthritis (OA) and rheumatoid arthritis (RA), are among the most prevalent disabling musculoskeletal disorders (1–3). Osteoarthritis, the most common form of arthritis, is characterized by the degradation of articular cartilage and subchondral bone, osteophytes formation and synovitis, and it is mainly induced by joint injury or overuse (3). On the other hand, RA is a systemic autoimmune disease that results primarily in inflammation and joint damage (4). It has been reported that not only RA and OA prevalence is higher in women, but women also report higher pain levels arising from these conditions than men (5). The mechanisms underlying these sex-related differences are still largely unknown. Psychological factors, body fat mass, sex hormones, and sex-related differences in immune response may account for this sex dimorphism (5). The available therapies do not prevent or revert the progression of these diseases, and in end-stage arthritis, joint replacement surgery is the gold standard procedure to provide pain relief and recover joint function (6, 7). Nevertheless, the slow progressive inflammatory response to the implant debris promotes periprosthetic osteolysis (PPOL) that may result in implant aseptic loosening (AL), a major threat to long-term implant survival (8).

Pain is a common symptom of RA, OA and AL, and has a great impact on the physical function and patients’ wellbeing (9, 10). Radiographic findings of joint degeneration do not always correlate positively with joint pain (11–13). This could be due to the fact that radiography imaging does not allow the assessment inflammatory changes (e.g. synovial hypertrophy and synovitis). To evaluate inflammation ultrasound and magnetic resonance imaging-based studies are needed. Inflammatory features are indeed positively and linearly correlated with joint pain (14, 15). These findings identify inflammation as an important predictive factor of pain.

The neuroimmune interplay has been demonstrated to have a key role in the generation and maintenance of pain in diseases with an inflammatory component (16). A diverse repertoire of inflammation-derived mediators can interact with nociceptors and modulate their activity. Among these mediators, pro-inflammatory cytokines, chemokines, growth factors and damage-associated molecular patterns (DAMPs- damaged molecules released from damaged or dying cells that bind to pattern recognition receptors [PRRs, e.g., toll-like receptors (TLRs)] (17)) have been shown to interact with receptors in the nociceptors, inducting their sensitization (18, 19). On the other hand, neuropeptides released by sensory nerve endings, such as Substance P (SP) and calcitonin gene-related peptide (CGRP), bind to their respective receptors located on the surface of immune cells, modulating their activity (20, 21). The sympathetic neurotransmitters, in particular the norepinephrine, are able to interfere with the inflammatory process, through the adrenoreceptors (ARs) expressed by immune cells (22).

Among the immune cells involved in the neuroimmune interplay, the macrophages have been highlighted as key players in the context of chronic inflammatory diseases (23). Macrophages express the receptors for the neurotransmitters and release mediators able to activate and sensitize nociceptors (24). Moreover, the targeting of macrophages can result in pain reduction (23).

In this narrative review, we discussed the mechanisms underlying the interaction between the inflammatory response and nerve fibers in RA, OA and AL regarding the genesis and maintenance of pain, in particular the role of macrophages was highlighted. The neuronal network and the immune response in joint health and disease are also described. Furthermore, the immunomodulation by the sensory and sympathetic neurotransmitters was revised. The role of neuroimmune interaction in pain has been addressed in recent reviews [e.g (25–30)]. Here, we focus instead on the neuroimmune crosstalk in pain associated to RA, OA, and AL diseases.

## 2 The Inflammatory Response in Joint Disorders

Although the severity of the immune response is higher in RA and AL, the involvement of an inflammatory component in the OA pathology is also well recognized. Here, the most relevant inflammatory processes in these joint disorders are described.

### 2.1 Synovial-Resident Macrophages

**Figure 1:**
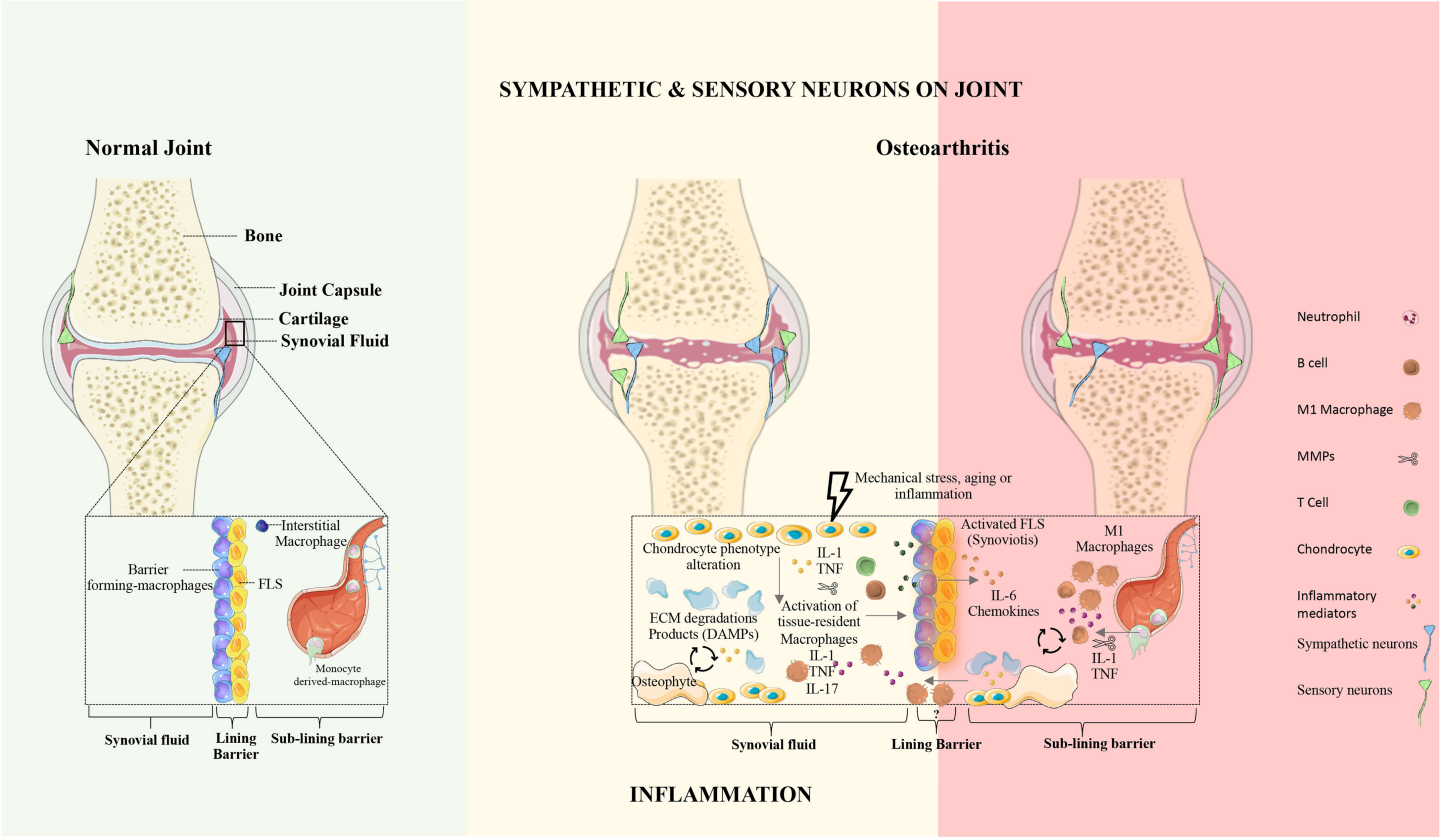
The synovial joint in health and osteoarthritis. Normal joint: In the healthy joint, the synovium is formed by two layers, the lining and the sub-lining. The lining layer is composed of barrier-forming macrophages and fibroblast-like synoviocytes (FLS). The sub-lining layer is composed of fibroblasts, tissue-resident macrophages, nerves, and blood vessels. In physiologic conditions, the articular cartilage is not innervated and does not contain blood vessels. Osteoarthritis: OA is characterized by articular damage and osteophyte formation. The presence of specific cell subsets and inflammatory mechanisms in OA remains unclear. In OA the innervation profile is closely linked to inflammatory severity. FLS, fibroblasts-like synoviocytes; ECM, extracellular matrix; DAMPs, damage-associated molecular patterns; IL, Interleukin; TNF, Tumor necrosis factor.

Synovial joints are characterized by the presence of a fluid-filled joint cavity surrounded by the synovium. The synovium is formed by two layers, the lining and the sub-lining. The lining layer is composed of tissue-resident macrophages and fibroblasts (31). The sub-lining layer is formed by fibroblasts, tissue-resident macrophages, nerves, and blood and lymphatic vessels (31). The disruption of synovial membrane architecture occurs early in joint inflammation (32). Culemann et al. (33) demonstrated that macrophages in the lining layer protect joints from inflammatory immune-cell assaults that are associated with arthritis. The authors studied the origin, differentiation, and distribution of these macrophages during steady-state and arthritis. The barrier-forming macrophages consist of a distinct population of CX3C chemokine receptor 1-positive (CX_3_CR1^+^) tissue-resident macrophages and derive from interstitial macrophages CX_3_CR1^-^ in the sub-lining layer (**Figure 1**) (33). Interestingly, these barrier-forming macrophages express tight junction proteins like the epithelial cells and provide an anti-inflammatory barrier around the joint that is disrupted during arthritis (33). Non-resident macrophages that infiltrate the joint are central effectors of synovial inflammation and arise from circulating monocytes (33).

Healthy synovial fluid lacks immune cell trafficking. Joint inflammation occurs when leukocytes infiltrate the synovial compartment. The severity of joint inflammation correlates with the degree of synovial macrophages infiltration, and depletion of these macrophages has a profound therapeutic benefit (34).

Macrophages are specialized phagocytic (scavenger-like) cells that recognize, ingest, and degrade cellular debris and pathogens. They act both in innate and adaptive immunity and are essential for the maintenance of tissue homeostasis and tissue repair processes. Recently, it was reported that macrophages derived from two main cellular lineages. One lineage emerges from bone-marrow-derived immune cells, the monocytes. The other lineage is monocyte independent, seeded in the tissues during embryonic development, and derived from the yolk sac, fetal liver, and bone marrow (35). Tissue-resident macrophages present a distinctive gene-expression profile, defined by the local microenvironment (36, 37). Adding to this complex origin, macrophages are very plastic cells and are often described as exhibiting a pro-inflammatory phenotype (M1 macrophages) or an anti-inflammatory more prone to tissue repair phenotype (M2 macrophages). Induced by the type 1 helper (Th1) cells signature cytokine (interferon (INF)-γ and tumor necrosis factor (TNF)-α) and/or the TLRs ligands (e.g. LPS), M1 macrophages secrete pro-inflammatory cytokines and low levels of interleukin (IL)-10. M2 macrophages result from the stimulation of Th2 signature cytokine IL-4 and/or IL-13 and secrete IL-10 and transforming growth factor (TGF)-β (38–40).

### 2.2 The Inflammatory Response in Osteoarthritis

Osteoarthritis is a degenerative disease characterized by articular cartilage destruction, subchondral bone remodeling, synovitis, and osteophyte formation (41) (**Figure 1**), with higher prevalence in females. Zhu et al. recently demonstrated a strong relationship between the increased levels of proinflammatory cytokines (IL-17F and IL-23) and bone marrow lesions in females but not in males. Therefore, it is critical to unveil the mechanisms underlying sex-related differences. Further studies regarding inflammatory mediators in OA should be performed on animal models of both sexes (42).

Osteoarthritis synovium shows hyperplasia, with an increased number of lining cells and a mixed inflammatory infiltrate. Macrophages are the most abundant immune cell type in the synovium, and their accumulation in the intimal lining is the first signal of synovitis (43). Kraus VB et al. provided the first study in humans demonstrating the involvement of macrophages in OA. Using Single photon emission computed tomography- Non-contrast (SPECT-CT) imaging, after the injection of Etarfolatide, which detects activated macrophages *in vivo*, the authors observed a correlation between the intensity of Etarfolatide uptake with radiographic severity of joint shrinking and osteophyte formation (44). Activated macrophages release inflammatory mediators, namely TNF-α and IL-1β, that alter the function of synovial fibroblasts and chondrocytes, leading to an increase in inflammation and catabolism in the joints (45). During cartilage degradation, DAMPs released from the extra-cellular matrix (ECM) to the joint space, signal to macrophages to trigger a protective response and eventually lead to repair (17). However, prolonged or dysregulated activation by DAMPs can be destructive and it has been implicated in the perpetuation of the low-grade systemic inflammation observed in OA, by the activation of the inflammasome pathways in macrophages (46). It was recently reported that mouse macrophages that were exposed to cartilage fragments, significantly upregulated genes involved in scavenger activity [phagocytosis, macrophage receptor with collagenous structure (Marco)], integrin-binding activity (migration, Itga5), TNF signaling and TLR signaling (TLR2). These results were further confirmed by immunohistochemical analysis of the synovial tissues obtained from OA patients. Results from RNAs-seq analysis of murine macrophages revealed the expression of MARCO, TLR2 and ITGα5 in the intima lining layer of synovial tissues. Importantly, their inhibition with specific antibodies significantly decreased the release of TNF-α by macrophages stimulated with cartilage byproducts (47). To decode the role of the different joint tissues in synovitis, Belluzi E. et al., cultured the human fibroblasts-like synoviocytes cell line K4IM in the presence of conditioned media (CM) from cartilage, synovial membrane, meniscus and infrapatellar fat pad (IFP) obtained from tissues of patients facing total knee arthroplasty. All the conditions tested led to the production of high levels of IL-6, IL-8, and C-C Motif Chemokine Ligand (CCL2). However, only K4IM cells cultured with CM from OA synovium demonstrated an increase of Il-6, CXCL8, metalloproteinase (MMP)10 and IL-1β expression. These results showed that in OA all joint tissues played a role in the progression of synovial inflammation, in particular the synovial membrane (48). The new findings of synovial macrophage subsets in RA that go beyond the classical M1/M2 concept, could potentiate the development of new therapeutic approaches that promote the resolution of synovial inflammation in OA. The presence of the same cellular subtypes and mechanisms in OA remains unclear and it is under investigation.

### 2.3 The Inflammatory Response in Rheumatoid Arthritis

**Figure 2:**
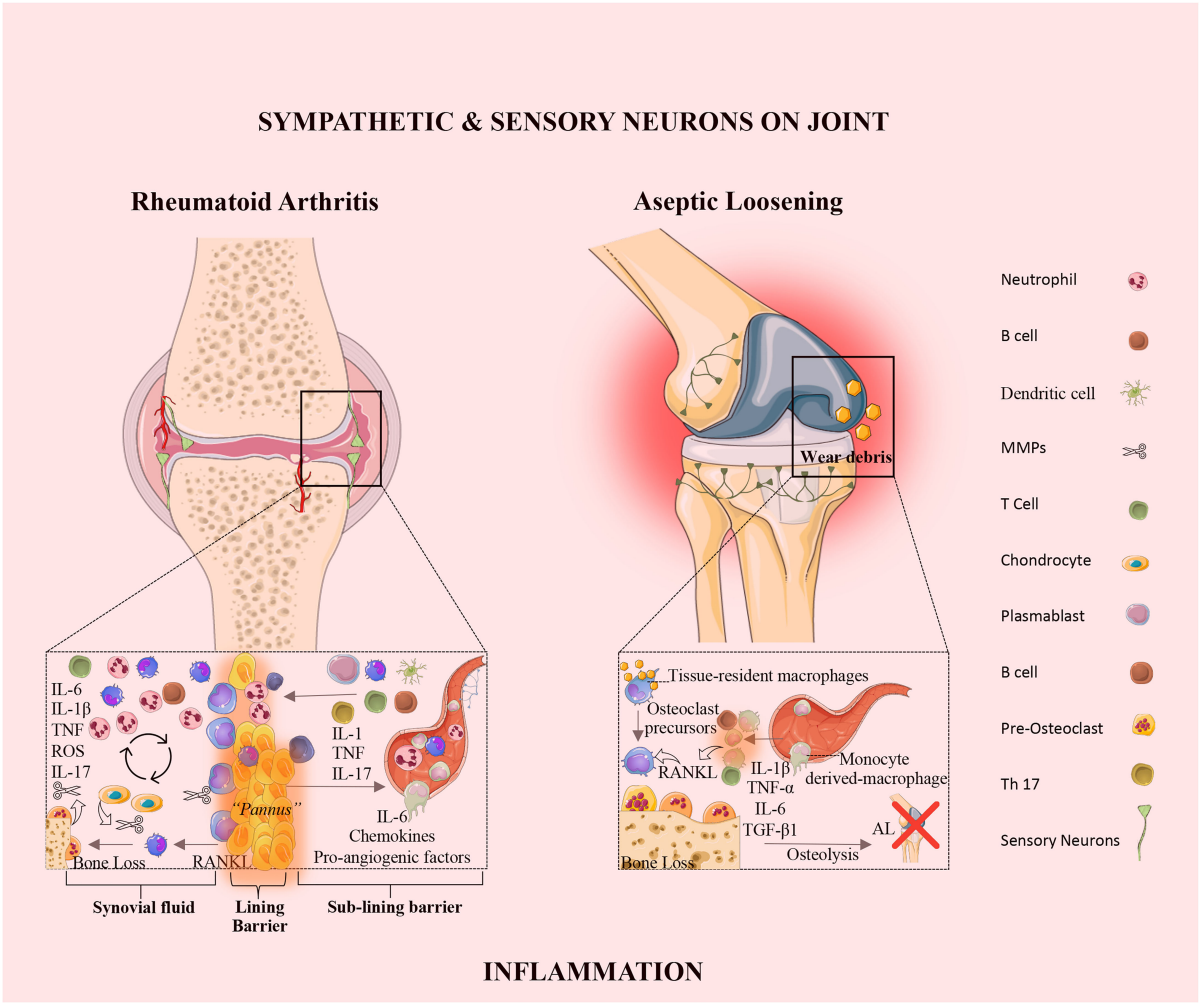
The synovial joint in rheumatoid arthritis and aseptic loosening. Rheumatoid Arthritis: In RA, the synovial intimal lining expands and forms an invasive hyperplastic pannus and sensory innervation increases, and sympathetic innervation is lost. The vicious cycle mediated by the interactions shown between immune cells, synovial fibroblasts, chondrocytes, and osteoclasts, together with the molecular products of damage, drive the chronic phase in the pathogenesis of RA. Aseptic loosening: In AL, wear particles released from implant devices and accumulated around the bone-implant interface, induce the release of pro-inflammatory mediators by tissue-resident macrophage. The innervation profile in AL is characterized by the rearrangement of sensory neurons and the absence of sympathetic fibers. IL, interleukin; TNF, tumor necrosis factor; ROS, reactive oxygen species; RANKL, receptor activator of nuclear factor kappa-B ligand; TGF, transforming growth factor; AL, aseptic loosening; Th, T-helper.

Rheumatoid arthritis is an autoimmune disease, characterized by synovial inflammation and hyperplasia, the production of autoantibodies (i.e., antibodies that react to self-antigens) to immunoglobulin G [IgG; rheumatoid factor (RF)] and citrullinated proteins [anti-citrullinated proteins antibodies (ACPAs)], and joint destruction. Synovial inflammation is the dominant feature in the early stage of RA (49). The pathology of RA is also characterized by the proliferation and activation of synovial tissue fibroblasts (50), as well as the trafficking of key cellular subsets, namely neutrophils, CD4^+^ T cells (51), B cells (52), and monocytes/macrophages (53) into the synovium, giving rise to an invasive tissue or *pannus* (**Figure 2** and **Table 1**). These interactions promote the release of several inflammatory mediators, including TNF-α, IL-1β, IL-6, IL-17, IL-23, IL-10, and TGF-β, which directly affect cartilage and bone cells, thus perpetuating the inflammatory cascade (**Figure 2** and **Table 1**).

**Table 1:** Contribution of different cell types to Rheumatoid Arthritis.

Cell Type	Subtype	Role in RA	Trigger factors in RA	Target and Action	Ref.
Synovial Fibroblasts		Maintain the inflammation, support the recruitment, survival and accumulation of leukocyte populations in synovium. Ostoclastogenesis.	CXCL8, CCL2, CCL5, CXCL10, CXCL5, CXCL1 and MMPs	JAK inhibitors suppressed the inflammatory response induced by oncostatin M in RA synovial fibroblasts.	(50, 54–56)
				In mice, genetic removal or blockade of NOTCH3 signaling decreases inflammation and hampers joint damage in inflammatory arthritis.	(57)
	FAPα^+^THY1^+^ immune effector fibroblasts	Located in the synovial sub-lining. Little effect on bone and cartilage erosion. Mediators of severe and persistent inflammation.	IL-6, IL-33, IL-34	In mice, deletion of FAPα^+^ fibroblasts impair inflammation and bone erosions.	(58)
	FAPα^+^THY1^−^ destructive fibroblasts	Located in the synovial lining layer.Bone and cartilage damage.	CCL9, TNFSF11, MMP3, MMP9, MMP13, RANKL		
B cells		Antibody producer, APC, T Cell activation, cytokine producer, osteoclastogenesis.	Receptor activator nuclear factor kappa-B ligand (RANKL), IL-1, IL-6, TNF-α	In patients, B cell depletion, with the use of rituximab provided significant improvements in disease symptoms.	(59–63)
T Cells	Th-1	Cytokine producer, macrophage activation.	IFN-γ, IL-2, TNF-α	Direct inhibition of T cells by or T cell depletion has exhibited limited or no therapeutic efficacy.	(64, 65)
	Th-2	Cytokine producer, B-cell activation.	IL-4, IL-5		(66)
	Th-17	Cytokine producer, MMPs stimulation, promote pannus growth, neoangiogenesis and osteoclastogenesis.	IL-17A, IL-17,F, IL-21, IL-22, TNF-α		(67)
	Treg	Suppress autoreactive lymphocytes, immunoregulatory functions.	IL-10, TGF-β		(68, 69)
	Th9	Neutrophils survival and Th17 differentiation.	IL-9		(70, 71)
	Tph	B cells activation.	IL-21		(72)
DCs		APC, T cells activation.	CXCL8, CCL3, CXCL10, IL-1β, IL-6, IL-12, IL-23		(73–78)
Neutrophils		First responders, cytokine producers. Involved in the earliest phase of arthritis.	TNF-α,IL-1β, IL-6, IL-8, LTB4 and CXCL5	Neutrophils depletion impairs arthritis development.	(79–84)
				PAD4 expression mediated by IL-6 was recently implicated in RA onset.	(85)
Macrophages	M1	Phagocytic cells, Present in high number in RA.M1>M2 ratio.	CXCL7, IL-1, TNF-α	Targeting inflammatory macrophages inducing their apoptosis, has a therapeutic benefit in reducing joint inflammation.	(34, 86–93)
	M2		IL-10	IL-10 deficient mouse model showed that the lack of IL-10 aggravated the inflammatory arthritis phenotype.	(94)
	AtoMs	AtoMs are the osteoclast precursor-containing population in the pannus tissue	RANKL	Tamoxifen inhibited the capacity of AtoMs to differentiate into osteoclasts *in vitro* and *in vivo*.	(95)
	CX3CR1^+^ macrophages	Barrier-forming CX3CR1^+^ macrophages; Maintenance and protection of the joints against inflammation	N/A	In arthritis, barrier-forming macrophages layer rapidly disintegrated and cells loosened their physical interactions, accelerating neutrophil influx.	(33)
	IL-1β^+^HBEGF^+^ macrophages	Pro-inflammatory tissue-damaging profile. HBEGF^+^ macrophages promote fibroblast invasiveness	IL-1 and EGF	HBEGF^+^ macrophages promote fibroblast invasiveness through an epidermal growth factor receptor.	(96)

APC, Antigen presenting cell; AtoMs, Arthritis-associated osteoclastogenic macrophages; CCL2, Chemokine (C-C motif) ligand 2; CCL3, Chemokine (C-C motif) ligand 3; CCL5, Chemokine (C-C motif) ligand 5; CCL9, Chemokine (C-C motif) ligand 9; CXCL1, C-X-C Motif Chemokine Ligand 1; CXCL10, C-X-C Motif Chemokine Ligand 10; CXCL5, C-X-C Motif Chemokine Ligand 5; CXCL7, C-X-C Motif Chemokine Ligand 7; CXCL8, C-X-C Motif Chemokine Ligand 8; DC, Dendritic cell; EGF, Epithelial growth factor; FAPα, Fibroblast activation protein-α; HBEGF, Heparin Binding EGF Like Growth Factor; IFN-, Interferon gamma; IL-1, Interleukin 1 beta; IL-12, Interleukin 12; IL-17, Interleukin 17; IL,-7A, Interleukin 17 A; IL-2, Interleukin 2; IL-21, Interleukin 21; IL-22, Interleukin 22; IL-23, Interleukin 23; IL-33, Interleukin 33; IL-34, Interleukin 34; IL-4, Interleukin 4; IL-5, Interleukin 5; IL-6, Interleukin 6; LTB4, Leukotriene B4; M1, Pro-inflammatory macrophages; M2, Anti-inflammatory macrophages; MMP13, Matrix metalloproteinases 13; MMP3; Matrix metalloproteinases 3; MMP9, Matrix metalloproteinases 9; MMPs, Matrix metalloproteinases; PAD4, Protein Arginine Deiminase; RA, Rheumatoid arthritis; RANKL, Receptor activator of nuclear factor kappa-B ligand; TGF-, Transforming Growth factor beta; Th17, T helper 17 cells; Th9, T helper 9 cells; THY1, Thymocyte differentiation antigen 1; TNF-, Tumor necrosis factor alpha; TNFSF11, Tumor necrosis factor ligand superfamily member 11; Tph, T peripheral helper cells; N/A, not applicable.

### 2.4 The Inflammatory Response in Aseptic Loosening

The molecular mechanisms responsible for PPOL/AL are still elusive. Tissue inflammation in response to prosthetic byproducts has been pointed to as the main responsible for AL (97). Wear particles released from implant devices that accumulate around the bone-implant interface, induce the release of pro-inflammatory mediators by tissue-resident macrophages (98) (**Figure 2**). The continuous release of wear particles perpetuates a chronic low-grade inflammation around the implant. This low-grade inflammation promotes the secretion of osteoclastogenic mediators, namely RANKL and IL-1β, which induce the differentiation of macrophages into osteoclasts (99), leading to bone resorption. Vasconcelos DM et al. analyzed the local immune profile and inflammatory response in AL and compared it to OA. The authors concluded that AL and OA differ in tissue architecture, immune cell distribution and local TGF-β1 expression. The results revealed that in OA the inflammation is restricted to the synovial membrane, in turn in AL, macrophages invaded all the periprosthetic tissue. In this study wear particles on aseptic interface tissues did not induce local macrophage polarization (M1 or M2), receptors from both profiles were present and evenly distributed in macrophages. In addition, AL and OA share similar systemic profiles in mRNA levels of the pro-inflammatory makers, namely TNF-α, IL-1β, IL-6, iNOS and RANKL. Interestingly, an increase of TGF-β1 expression in tissues from AL patients was observed when compared to OA. These results suggest a relation between TGF-β1 expression and the immune response in AL and OA (100). Recently, Dyskova et al. in a pilot study analyzed the inflammation protein signatures in tissues collected from patients with failed total knee arthroscopy due to AL. The authors found that AL tissues presented high levels of TNF-family members [sTNFR2, TNFSF14, sFasL, B cell activating factor belonging to the TNF family (sBAFF)], cytokines and chemokines (IL,8, CCL2, IL1RA/IL36, sIL6R), and growth factors [amphiregulin (sAREG), colony stimulating factor (CSF1)]. Macrophages and osteoclast-like cells are the producers of these inflammatory molecules in AL (101). The sTNFR2 in AL was already reported as having a role in the osteoclast formation, and the absence of sTNFR2 receptors impairs osteoclastogenesis (102). Further studies with a larger patient cohort and using single-cell transcriptomic analysis will be helpful to confirm and identify the key players in AL.

## 3 The Joint Innervation

The synovial joints are highly innervated by sensory and sympathetic nerve fibers. Changes in the innervation pattern are supposed to contribute to the generation and maintenance of arthritis pain. Here, the joint innervation pattern is described in physiologic and in arthritic conditions.

### 3.1 The Joint Innervation in Physiological Conditions

Unequivocal evidence has demonstrated the innervation of the capsule, ligaments, synovium, meniscus, fatpads, subchondral bone and periosteum in the synovial joints (103–106). In contrast, in physiologic conditions, articular cartilage is not innervated and does not contain blood vessels (105, 106).

The synovial joints are innervated by sensory thinly myelinated A-δ fibers and unmyelinated peptide-rich C-fibers. The A-δ fibers are mainly present in the capsule, ligaments and meniscus (107). These nerve fibers release the neuropeptides CGRP and SP (108, 109). They are mostly sensitive to nerve growth factor (NGF) and express the tropomyosin receptor kinase A (TrkA) (108, 109). Typically, the A-δ fibers are sensitive to high threshold stimulation, and respond to noxious mechanical stimuli and in a lesser extent to thermal stimuli (108, 109). The unmyelinated C-fibers are the most abundant nerve fibers in the synovial joints (107). As the A-δ fibers, the majority of these C-fiber are peptidergic, releasing SP and CGRP upon activation, and are also TrkA-positive (108, 109). The C-fibers also have a high threshold, and they respond to multi stimuli modalities, such as mechanical, thermal, and chemical stimuli (108, 109). The innervation of joints by A-β myelinated fibers is scarce and mostly restricted to the capsule and ligament surface (107). They have very low thresholds and are sensitive to mechanical stimulation (108, 109). Overall, the joint sensory innervation is mostly composed of silent nociceptors (the C-fibers and the A-δ fibers), which respond only to potentially dangerous stimulation.

Sympathetic nerve fibers innervate the synovial joint structures, except for the articular cartilage (110). These nerve fibers express tyrosine hydroxylase (TH, the rate-limiting enzyme for biosynthesis of catecholamines) and release norepinephrine, and are mostly associated with blood vessels (31). The sympathetic nerves express TrkA, thus they are also influenced by NGF (111). The sympathetic neurotransmitters can modulate indirectly joint pain due to their vascular- and immune-regulatory action, but also through the direct modulation of nociceptors activity (112).

### 3.2 Alterations in Joint Innervation Pattern in Joint Disorders

Alteration in the pattern of innervation has been described in several pathological conditions. The sprouting of sensory and sympathetic innervation was associated with pain in non-healing bone fractures (113). The sprouting of sensory nerve fibers was also described in bone cancer pain and chronic discogenic pain (114, 115), and the sprouting of sympathetic nerve fiber in neuropathic pain (116). In contrast, repulsion of the sympathetic innervation has been reported in severe chronic inflammatory conditions, such as Crohn’s disease and diabetes (117, 118). Alterations in the innervation pattern have also been described in joint disorders and associated with the severity of pain.

In OA, data from animal models and human studies revealed alterations of both sensory and sympathetic innervation profile. In the OA mouse model induced by the destabilization of the medial meniscus (DMM), an increase in the sensory nerve fibers was observed in the medial synovium, meniscus, and subchondral bone at 6 weeks after OA induction (119). In the same mouse model, our group reported no changes in the sensory innervation of the synovium, meniscus, or subchondral bone 12 weeks after surgery, however, the sensory innervation increased in the periosteum (120). The difference between the two studies might be related to the disease progression stage. In the monoiodacetate (MIA)-induced OA rat model, sprouting of CGRP and TrkA positive fibers was observed in the knee joint (121). The increase in the joint innervation was observed also in human samples. Suri et al. (122). reported that sensory and sympathetic nerve fibers were observed in articular cartilage, following the neovascularization breaching the tidemark, in human samples of the knee with mild and severe OA (122).

Studies in RA animal models also revealed alteration in the joint innervation. The density of sensory (CGRP^+^, NF200^+^) and sympathetic (TH^+^) nerve fibers was increased in the synovium in the Complete Freund’s Adjuvant (CFA)-induced mouse model (123). Interestingly, early and sustained administration of anti-NGF therapy prevented the sprouting of the sensory and sympathetic nerve fibers and reduced the pain-related behaviors in this model, supporting the involvement of nerve sprouting in arthritic pain (123). The sensory (CGRP^+^) and sympathetic (VMAT-2^+^) nerve fibers density was also increased in the synovium of the ankle joint in CFA-induced rat model (124). The inhibition of sympathetic signaling with guanethidine reduced pain-related behaviors, supporting its involvement in joint pain (124). In contrast, a decrease in the sensory innervation of the synovial membrane was observed in the inflammatory CIA mouse model (125). A decrease of the sensory nerve fibers in the synovium was also reported in human OA, and correlated with inflammation (126). This effect may be related to a high degree of inflammation. Further investigation is needed to clarify the putative link between the alteration in the joint innervation profile and the inflammation.

Studies with samples from RA patients reported a loss of sympathetic innervation (127–129) and the magnitude of the sympathetic nerve loss seems to correlate with the degree of inflammation. In fact, comparative studies between RA and OA described the loss of TH^+^ nerve fibers in the synovial tissue of RA patients but not in OA patients (127, 129). In contrast, a higher number of sensory nerve fibers (SP^+^) were detected in the synovial tissues from RA patients when compared to synovial tissues from OA patients (127, 129).

Similarly, our group has reported the absence of sympathetic nerve fibers (TH^+^) in periprosthetic tissues from AL patients, while this was not observed in OA patients (100). This further supports the results reported by Niissallo et al. showing the absence of sympathetic and C-peptidergic nerve fibers from synovial-like membrane [i.e., tissue surrounding the joint implant that has the histological and histochemical characteristics of a synovial-like lining (130)] (131). As described for RA, the loss of TH^+^ fibers seems to correlate with the severity of the inflammatory response. In contrast to Niissalo et al., Ahmed et al. showed that the synovial-like membrane from AL hip prostheses is supplied by sensory nerve fibers positive to SP, neurokinin A (NKA) and CGRP (132). Recently, we also reported the presence of sensory nerve fibers in this tissue (100). However, the organization of the sensory nerve fibers in synovial-like membrane was different from the organization in the synovial membrane in OA patients (100). SP^+^ and CGRP^+^ nerve fibers were observed in subintima regions of the synovial membrane mainly around blood vessels, whereas these nerve fibers were dispersed through the synovial-like membrane (100). In **Figure 1** the overall alteration in the innervation pattern are represented.

Although there is still much to know regarding joint innervation, it is clear that under pathological conditions joint innervation undergoes important reorganization that will impact pain transmission.

## 4 The Neuroimmune Crosstalk in Joint Disorders

The bidirectional communication between the peripheral nervous system and the inflammatory process is recognized to play a critical role in the generation and maintenance of pain (16). In response to noxious stimuli, nociceptors, adding to the generation of action potentials that will be transmitted to the spinal cord, release inflammatory mediators that promote the recruitment of immune cells and modulate their activity (133). On the other hand, the molecules released by the immune cells, such as cytokines, chemokines, lipid mediators and growth factors, will activate nociceptors evoking a pain response (18, 19). In this review, the neuroimmune crosstalk in the context of RA, OA and AL will be discussed.

### 4.1 The Role of the Inflammatory Mediators on the Nociceptor Activity and Evoked Pain

A diverse repertoire of inflammatory mediators, including cytokines, chemokines and DAMPs, are present at the arthritic joint, and able to activate the nociceptors initiating pain signaling (134, 135).

In OA, DAMPs have been suggested to be involved in the activation and sensitization of nociceptors, in particular by signaling through the TLR4 (136). The TLRs and the receptor for advanced glycation end products (RAGE) compose the class of receptors that recognize DAMPs and pathogen-associated molecular pattern (PAMPs), the pattern recognition receptors (PRRs), which are expressed also in nociceptors (137, 138). The blockade of TLR4 inhibits the release of MCP-1 induced by activation with S100A8 and α2 -macroglobulin (DAMPs molecules) in mouse dorsal root ganglia (DRG) *in vitro* cultures (138). Nevertheless, the deletion of TLR4 was not sufficient to suppress mechanical allodynia in the DMM mouse model (138). Although TLR4 seems to be involved in OA pain, alone its inhibition is not sufficient to reduce pain sensitization. Interestingly, the expression of S100A8 and S100A9 was not increased in the DMM mouse model, while it was upregulated in samples of OA patients undergoing joint arthroplasty and in the CIA mouse model (139). Moreover, the inhibition of TLR4 by the antagonist TLR4-A1 significantly reduced mechanical allodynia in the MIA rat model (140).

Although TLR4 is the most studied TLRs regarding pain (141), nociceptors express other TLRs, such as TLR2, TLR3, TLR4, TLR5, TLR7 and TLR 9 (141, 142). TLR2 was also shown to be involved in arthritic pain (143). In DRG cultures, the activation of TLR2 by stimulation with 32-mer was shown to induce the expression of the pro-algesic chemokine CCL2 (143). Moreover, the deletion of TLR2 prevented the development of knee hyperalgesia in the DMM mouse model of OA (143). Recently, the involvement of TLR7 activation in OA pain was also demonstrated (144). The algesic effects of miR-21 (miRNA highly released from synovial tissue in OA) are achieved through TLR7 activation (144). The antagonism of TLR7 blocked the miR-21-induced pain and had an analgesic effect in the Anterior Cruciate Ligament Transection (ACLT) OA rat model (144). The available data suggesting the involvements of TLRs in arthritic pain still needs further confirmation.

Pro-inflammatory cytokines such as TNF-α, IL-1β and IL-6 are released by chondrocytes, synoviocytes and infiltrating immune cells in joint disorders such as RA and OA (45, 145). Moreover, nociceptors express the receptors for these cytokines (134).

IL-1β increases the excitability of nociceptors through p38MAP kinase, by delaying the tetrodotoxin (TTX)-resistant voltage-gated sodium channels inactivation and increasing its current threshold (146). The long-term administration of diacerein, a powerful inhibitor of IL-1β synthesis, to patients with moderate to severe knee OA resulted in a significant reduction of pain (147, 148). Moreover, the intraarticular administration of anakinra, an IL-1 receptor antagonist, reduced pain and improved knee function in patients with an acute knee injury (149).

TNF-α is known to directly activate nociceptors through TNF receptor 1 (TNFR1), inducing the up-regulation of voltage-gated sodium channels (Nav1.3 and Nav1.8), and resulting in ectopic impulse and spontaneous discharge of nociceptors, leading to mechanical hyperalgesia (150). The concentration of TNF-α in the synovial fluid positively correlates with the pain intensity in patients with knee OA (151). The neutralization of TNF-α resulted in pain relief in patients with knee OA (152, 153) and antigen-induced arthritis (AIA) rat model (154).

IL-6 was also shown to play a role in the generation and maintenance of arthritic pain. In patients with knee OA, a positive correlation was found between the concentration of IL-6 in the synovial fluid and reported pain (155). The IL-6 signaling depends on the IL-6 binding to gp80 specific receptor (IL-6R) and then to the transmembrane signal-transducing subunit gp130 (156). Sensory neurons were shown to express the gp130 (157). The neutralization of IL-6/sIL-6R complexes by the intra-articular administration of sgp130, reduced the mechanical hyperalgesia during the acute phase of the AIA-arthritis rat model (158). In RA patients, anti-human IL-6 receptor antibody is being tested in clinical trials and show to result in an improvement of RA symptoms, including pain (155, 159).

IL-17 has been considered an important pro-inflammatory cytokine in RA (160), though its pro-nociceptive effects are less studied. The sensory neurons in the mouse DRG were shown to express all the IL-17 receptor subtypes (29). The deletion of IL-17A resulted in the decrease of mechanical hyperalgesia in the AIA mouse model, but it did not interfere with the disease severity (29). In OA, a correlation was found between the levels of IL-17 in the synovial fluid and pain in Knee OA patients (161). Overall, this data suggests a putative role of IL-17 in joint pain in RA, as well as in OA.

The chemokines, which are chemotactic cytokines, were also shown to be a key mediator in arthritic pain. A positive correlation was observed between the concentration of CCL2 in the synovial fluid and pain in knee OA patients (162, 163). The CCL2 receptor, the CCR2, is expressed in sensory neurons, and their activation was shown to sensitize nociceptors through the increase in the Na_v_1.8 Na^+^ currents and TRPV1 expression by the activation of the PI3K/Akt signaling pathway (164, 165). Moreover, it was shown that CCL2, which is also synthesized by DRG neurons, is involved in the DRG infiltration by macrophages, an important mechanism in arthritis pain. The deletion of CCL2 or CCR2 in mice delays the onset of pain-related behavior in the DMM OA mouse model (166), and the specific deletion of CCR2 inhibited the DRG macrophage infiltration (167).

As referred above, nerve growth factors also contribute to the mechanisms of chronic pain. The blockage of NGF has emerged as a putative analgesic therapy in several diseases, including in OA. The binding of NGF to TrkA increases the nociceptor excitability, by upregulating the expression of TRPV1, bradykinin receptors, purinergic P2X receptors and ion channels, and the synthesis of substance P and CGRP (168). The expression of NGF is known to be upregulated at the inflammatory sites (169). In the DMM OA mouse model, increased NGF mRNA levels were found in the knee joints, and the inhibition of NGF signaling reduced the mechanical hyperalgesia (170). Additionally, a single systemic administration of the anti-NGF antibody AS2886401-00 resulted in a long-term decrease in the gait deficit in the MIA-induced arthritic rat model (171). Additionally, the mRNA expression of TrkA was found to be upregulated in the DRG in the MIA rat model, which was reversed with the pre-treatment with indomethacin (nonsteroidal anti-inflammatory drugs (NSAIDs) (172). Moreover, the pre-treatment with indomethacin attenuated NGF-facilitated weight-bearing asymmetry (172). Promising results are coming from ongoing clinical trials with OA patients, in which few administrations of a monoclonal Anti-NGF antibody induce sustained pain relief in moderate to severe OA (173–175). Although issues regarding adverse effects related to the worsening of OA condition led to the interruption of the first clinical trials (176), permission was given to restart these studies.

Autoimmune mechanisms are also suggested to be involved in chronic pain (177). Autoantibodies were identified as molecules that can play a role in the neuroimmune interplay in pain in RA. These molecules were detected in RA patients [e.g. RF and ACPA (178)] and are able to increase nociceptors hyperexcitability, inducing pain (179). The autoantibodies activate the complement system, increasing the inflammatory response and indirectly promoting nociception (177). On the other hand, autoantibodies also interact directly with the nociceptors through binding to the Fc gamma receptors and disrupting the ion channels (177). The injection of ACPA was shown to induce nociception in mice (180). Moreover, it was demonstrated that this effect was associated with the IL-8 produced by the osteoclasts when stimulated by ACPA (180).

Although OA is not considered an autoimmune disease, autoantibodies were also detected in the serum and synovial fluid of OA patients (181), but no relationship between these autoantibodies and OA pain was yet described. Further studies are needed to clarify the role of autoantibodies in RA and OA.

#### 4.1.1 The Role of Macrophages on the Nociceptor Activity and Evoked Pain

The immune system has not always a detrimental role in pain. Although uncontrolled or unresolved acute inflammation evolves to chronic inflammation, which can lead to chronic inflammatory pain (182), the acute inflammatory response has a protective role, promoting tissue repair, and elimination of invading organisms. Macrophages have a pivotal role in acute inflammation and its resolution (183). These cells release mediators, such as maresins (MaRs), that have anti-inflammatory, analgesic and pro-resolving properties (184, 185). Maresins enhance macrophage phagocytosis, and promote the shift of cytokine release to the anti-inflammatory M2 phenotypes, downregulating proinflammatory cytokines (e.g., IL-1β, IL-6, and TNF-α) and increasing the synthesis of anti-inflammatory cytokines (IL-10 and TGF-β) (183). Specifically, macrophages were shown to promote analgesia *via* a mechanism dependent on IL-10 signaling in DRG (186). Additionally, the stimulation of M2 macrophages by IL-4 induces the synthesis of opioid peptides [e.g., Met-enkephalin, β-endorphin, and dynorphin A-(1-17)] that bind to the peripheral opioid.

In joint chronic inflammation, macrophages are known to be proinflammatory mediators, such as IL-1β, IL-6, and TNF-α, as well as NGF and CCL2 (24).

In a previous study, we identified macrophages as the largely dominant infiltrating cell population in the synovial membrane of OA patients and in the synovial-like membrane of AL patients (100). Moreover, SPECT-CT imaging analyses revealed a positive correlation between the number of activated macrophages in the knee and the severity of pain in patients with knee OA (44). Also, in patients with knee OA, the synovial and plasma levels of the CD14 (macrophages marker) correlate positively with pain (187). The pivotal role of macrophages has also been demonstrated in the development of RA (86–88).

Experiments targeting the macrophages confirm the involvement of these cells in the mechanisms of pain in joint disorders. In a rat model of advanced knee arthritis (produced by intra-articular injection of MIA), the depletion of synovial macrophage, *via* injection of clodronate liposomes, reduced the levels of IL-1β and NGF in the joint, and lead to the suppression of pain (188). It was also shown that the reduction in pain observed after the deletion of CCL2 and CCR2 in the DMM OA mouse model was associated with the suppression of joint levels of CD68 mRNA (macrophage marker), suggesting a role for the CD68 macrophages in the pain development (166). Moreover, the involvement of granulocyte-macrophage colony stimulating factor (GM-CSF) in pain in the CIA mouse model (189) was shown to be achieved *via* macrophages, which in response to GM-CSF synthesize mediators (CCL17, CCL22, CCR2, IL4ra, Irf4, Nfil3, Socs1, Socs2, and Socs3) that activate the nociceptors (190). This was further confirmed in a study performed in a GM-CSF-dependent CIA mouse model, showing that the neutralization of CCL17 reduces pain, and that the macrophages were the only cells in the synovium expressing CCL17 mRNA (191).

In several pain models, including models of RA and OA, the DRG infiltration by macrophages was identified as a mechanism of pain. A substantial increase in macrophages was reported bilaterally in knee-innervating lumbar level DRG in the AIA rat model, and this coincided with an increase in the expression of vascular cell adhesion molecule-1 (VCAM-1), a molecule involved in macrophage infiltration (192). Moreover, the inhibition of TNF-α reduced the lumbar DRG infiltration by macrophages and the VCAM-1 expression, as well as the mechanical hyperalgesia (192). In the DMM OA mouse model, an increase in the sensory neuron expression of CCL2 and CCR2 was coincident with the increase in the DRG infiltration by macrophages (167). The deletion of CCR2 was also shown to inhibit the DRG macrophage infiltration and reduce pain-related behaviors (166, 167). Recently, Raoof et al. (193) reported in rat models of OA (MIA and Groove surgery induced OA), that DRG macrophages display an M1-like proinflammatory phenotype, and systemic or local depletion of DRG macrophages reduce OA pain without affecting the pathology (193). Moreover, the inhibition of M1-like macrophages in DRG through intrathecal administration of an IL4-IL10 fusion protein or M2-like macrophages also reduced OA pain (193).

**Figure 3:**
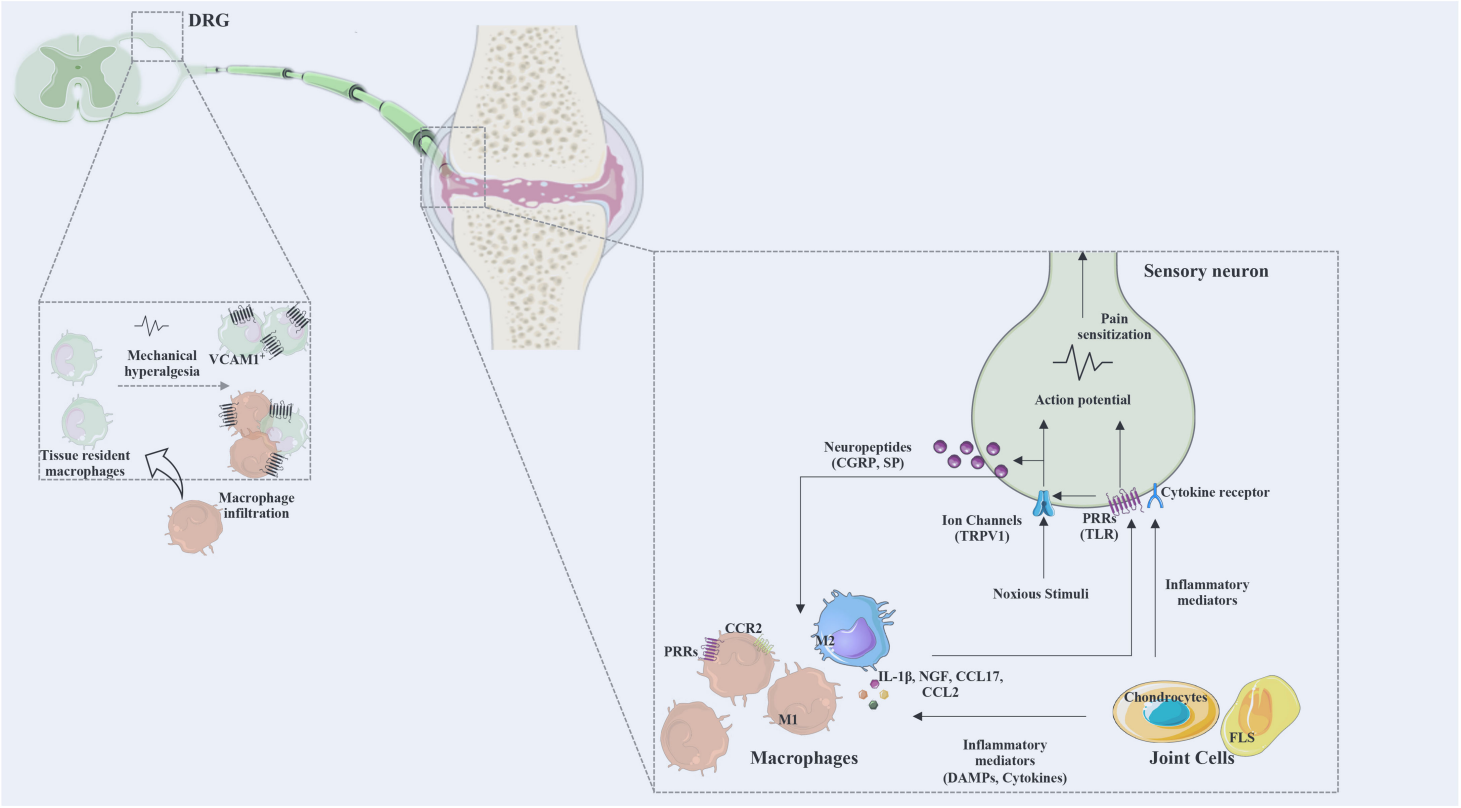
The interaction between macrophages and sensory neurons. Macrophages and joint cells release mediators (cytokines, chemokines, DAMPs, lipid mediators, growth factors) and noxious stimuli that act on ion channels and various receptors for these mediators. Action potentials are transduced via the dorsal root ganglia (DRG) to the spinal cord and transmitted to the brain to be processed as pain. Activated neurons release neuropeptides affecting immune cells and regulating inflammatory responses. DRG infiltration by macrophages was identified as a mechanism of pain. M1, classically activated macrophages; M2, alternatively activated macrophages; CGRP, calcitonin gene-related peptide; SP, substance P; PRRs, pattern recognition receptors; TRPV1, transient receptor potential cation channel V member 1; Na 1.7, voltage-gated sodium; TLR, Toll-like receptor; NGF, nerve growth factor; VCAM, vascular cell adhesion molecule; CX3CR, fractalkine receptor; DRG, dorsal root ganglia; CCR, chemokine receptor; CCL, C-C motif chemokine ligand; FLS, fibroblasts-like synoviocytes.

Overall, the described data provides evidence for the involvement of inflammatory mediators such as pro-inflammatory cytokines, chemokines and growth factors in the crosstalk between immune cells and nociceptors, and highlights macrophages as key cell players in this crosstalk. **Figure 3** summarizes the described mechanisms of interaction between macrophages and sensory nerve fibers.

### 4.2 The Role of Sympathetic and Sensory Neurotransmitters on the Inflammatory Response

The nerve fibers locally in inflamed tissues are known to modulate the inflammatory response. Activated nociceptors release into the periphery SP and CGRP, which will affect the inflammatory response. These neurotransmitters are strong vasodilator molecules and chemotactic for innate and adaptive immune cells, and they also modulate the activity of the immune cells (20, 21). Tewari et al. (190) showed that the conditioned medium from NGF-stimulated nociceptors modified macrophages gene transcription, upregulating the expression of inflammatory mediators and chemokines, such as IL-1β, IL6, and CCL22 (190). Moreover, it was shown, in an *in vitro* experiment, that sensory neurons that innervate the OA knee joint in the rat polarize DRG macrophages into an M1-like phenotype (193).

The binding of SP to the neurokinin-1 receptor expressed in macrophages, promotes an alteration in the macrophage phenotype towards a proinflammatory phenotype, as shown by the up-regulation of IL-1 and TNFα (194). On the other hand, evidence supports the anti-inflammatory role of CGRP (21). For instance, CGRP modulates TLR4-stimulated macrophages by enhancing the levels of regulatory cytokines, such as IL-10, through the activation of CREB-dependent gene transcription (195). Substance P and CGRP are known to be involved in the modulation of inflammatory response (20, 21), and specifically, evidence suggests their involvement in chronic joint inflammations. These neuropeptides were found to be upregulated in the synovial fluid of patients with OA and RA (196). Moreover, upregulate levels of these neuropeptides positively correlated with the levels of pro-inflammatory cytokines (197). The levels of SP and CGRP were upregulated in the pseudosynovial fluid of implant AL patients (198). The modulation of SP and CGRP might be a putative strategy to reduce inflammation in chronic joint inflammatory conditions, as demonstrated in other inflammatory conditions (199, 200).

Evidence has attributed a pivotal role to sympathetic signaling, in the regulation of chronic inflammatory conditions. The immunomodulatory effects of norepinephrine, the major peripheral catecholamine, can be achieved directly through the activation of the alpha (α) and beta (β) adrenergic receptors (ARs) expressed by the immune cells (22). The stimulation of β2-AR, responsive to high concentrations of norepinephrine, is reported to trigger anti-inflammatory mechanisms on immune cells, whereas the stimulation of β-AR, responsive to high concentrations of norepinephrine, activates pro-inflammatory mechanisms (22).

Studies inducing sympathectomy or the administration of β-AR agonist or antagonists in rodent adjuvant-induced arthritis or CIA models, revealed a dual role of the sympathetic nervous system (201). Proinflammatory effects of norepinephrine were observed at the early phase of the disease and anti-inflammatory effects at the later phase (202, 203). In another mouse model of RA, zymosan-induced arthritis, the administration of β-agonists at initial stages of the disease, induced anti-inflammatory effects, while the administration of α1- and α2-agonists induced either pro- and anti-inflammatory effects, respectively (204). The overall effect depends on the receptor expression profile and on the norepinephrine concentration.

Interestingly, the loss of sympathetic innervation in severely inflamed tissues has been reported (117, 118). In RA, data from animal models and human studies revealed the loss of sympathetic nerve fibers in the synovium (127–129). This absence was also reported in the synovial-like membrane of hip implant aseptic-loosening patients (100). Moreover, this effect is correlated with the harshness of the inflammatory response (127, 129). Comparative studies between RA and OA, and AL and OA, show the reduction of sympathetic innervation in the synovium of RA patients and the synovial like-membrane of AL patients, but not in the synovial membrane of OA patients (100, 127). In RA the loss of sympathetic might be compensated by the appearance of TH^+^ catecholamine-producing cells as observed in the CIA mouse model (128), but not in AL (205).

In implant AL, expression of β2-AR was also shown to be absent in the macrophages in synovial like-membrane of AL patients (205). The stimulation of β2-AR in macrophages induces a shift in their polarization towards an anti-inflammatory phenotype, which acts a way to prevent hyper-inflammation (206). Thus, the absence of β2-AR signaling in macrophages in AL suggest a preferential polarization of macrophages towards a proinflammatory phenotype, which could be underlying the severity of the inflammatory response and the consequent osteolysis. Overall, the local sympathetic nervous system emerges as a putative target to control the inflammatory response in RA and AL.

## 5 Conclusion

The neuroimmune crosstalk has been investigated in the context of joint disorders regarding chronic pain. The literature reviewed in this manuscript support that RA and OA pain share neuroimmune mechanism. Regarding the AL, the neuroimmune crosstalk remains poorly understood. The information gathered so far demonstrated the ability of inflammatory mediators, such as pro-inflammatory cytokines (IL-1β, TNF-α, IL-6, and IL-17), CCL2, and NGF, to interact with receptors in joint nociceptors, inducing their activation and sensitization in OA and RA. Moreover, DAMPs were also reported to be involved through the activation of TLRs expressed in the nociceptors. In this process, the macrophages emerged as the key immune cell player. Experiments targeting the macrophages confirm their involvement in the mechanisms of joint pain.

On the other hand, sensory neurotransmitters are known to modulate inflammatory activity. This immunomodulatory effect in joint disorders is supported by the increased expression levels of SP and CGRP in the synovial fluid of OA, RA, and AL patients. Interestingly, sympathetic immunomodulation seems to be reduced in severe inflammatory conditions like RA and AL. The alterations in the levels of the neurotransmitters positively correlate with inflammation.

The comprehensive understanding of the mechanisms underlying the neuroimmune crosstalk in the context of joint disorders will support the identification of novel therapeutic targets to treat the associated chronic pain.

## Author Contributions

DPV, CJ and CJA conceived and wrote the manuscript. DPV and CJ are co-first authors of the manuscript. ML contributed to original draft preparation. All authors participated in the revision of the manuscript, read, and approved the submitted version.

## Acknowledgments

The authors received funding from European Union’s Horizon 2020 research and innovation program under grant agreement No. 860462 project PREMUROSA.

## Conflict of Interest

The authors declare that the research was conducted in the absence of any commercial or financial relationships that could be construed as a potential conflict of interest.

## Publisher’s Note

All claims expressed in this article are solely those of the authors and do not necessarily represent those of their affiliated organizations, or those of the publisher, the editors and the reviewers. Any product that may be evaluated in this article, or claim that may be made by its manufacturer, is not guaranteed or endorsed by the publisher.

## Abbreviations

ACLT, Anterior Cruciate Ligament Transection; ACPAs, Anti-citrullinated proteins antibodies; AIA, Antigen-induced arthritis; α-AR, Alpha-adrenergic receptors; AREG, Amphiregulin; APC, Antigen presenting cell; ARs, Adrenoreceptors; β-AR, Beta-adrenergic receptors; BAFF, B cell activating factor belonging to the TNF family; CCL, Chemokine (C-C motif) ligand; CD 24, Cluster of differentiation 24; CFA, Complete Freund’s Adjuvant; CGRP, Calcitonin gene-related peptide; CIA, Collagen-induced arthritis; CM, Conditioned Medium; CSF1, Colony Stimulating Factor 1; CX3CR1, C-X3-C Motif Chemokine Receptor 1; CXCL, Chemokine (C-X-C motif) ligand; DAMPs, Damage-associated molecular pattern molecules; DC, Dendritic cell; DMM, Destabilization of the medial meniscus; DRG, Dorsal root ganglia; ECM, Extracellular matrix; EGF, Epidermal growth factor; FAPα, Fibroblast activation protein – alpha; FLIP, Flice Inhibitory Protein; GM-CSF, Granulocyte-macrophage colony stimulating factor; HLA-DR, Human Leukocyte Antigen – DR isotype; IFN-γ, Interferon gamma; IgG, Immunoglobulin G; IL-, Interleukin; iNOS, Inducible nitric oxide synthase; JAK, Janus kinase; LTB4, Leukotriene B4; MARCO, Macrophage receptor with collagenous structure; M-CSF, Macrophage colony stimulating factor; MIA, Monoiodacetate; MMPs, Matrix metalloproteinase; NF-κB, Nuclear factor kappa-light-chain-enhancer of activated B cells; NGF, Nerve growth factor; NKA, Neurokinin A; NSAIDS, Nonsteroidal anti-inflammatory drugs; OA, Osteoarthritis; P2X7, P2X purinoceptor 7; PAMPs, Pathogen-associated molecular patterns; PDCD, Programmed cell death protein 1; PPOL, Periprosthetic osteolysis; PTEN, Phosphatase and tensin homolog; RA, Rheumatoid arthritis; RAGE, Receptor for advanced glycation end-products; RANKL, Receptor activator of nuclear factor kappa-B ligand; RASFs, Rheumatoid arthritis synovial fibroblasts; RF, Rheumatoid Factor; SCID, Severe combined immunodeficiency; SEN-P1, Sentrin-specific protease 1; sFASL, soluble Fas Ligand; Siglec 10, Sialic Acid Binding Ig Like Lectin 10; sTNFR2, Soluble tumor necrosis factor receptor 2; SUMO-1, Small ubiquitin-like modifier-1; TFH, T follicular helper cells; TGF, Tumor growth factor; TH, Tyrosine hydroxylase; TIM-3, T cell immunoglobulin and mucin-domain containing-3; TLR, Toll-like receptor; TNFSF14, Tumor necrosis factor superfamily member 14; TNF-α, Tumor necrosis factor alpha; TPH, T peripheral helper cells; TRAP, Tartrate-resistant acid phosphatase; TREM1, Triggering receptor expressed on myeloid cells 1; TrKA, Tropomyosin receptor kinase A; TRPV1, Transient receptor potential cation channel V member 1; VCAM-1, Vascular cell adhesion molecule-1; VMAT-2, Vesicular monoamine transporter 2

## Glossary

**Table:**

ACLT	Anterior Cruciate Ligament Transection
ACPAs	Anti-citrullinated proteins antibodies
AIA	Antigen-induced arthritis
α-AR	Alpha-adrenergic receptors
AREG	Amphiregulin
APC	Antigen presenting cell
ARs	Adrenoreceptors
β-AR	Beta-adrenergic receptors
BAFF	B cell activating factor belonging to the TNF family
CCL	Chemokine (C-C motif) ligand
CD 24	Cluster of differentiation 24
CFA	Complete Freund’s Adjuvant
CGRP	Calcitonin gene-related peptide
CIA	Collagen-induced arthritis
CM	Conditioned Medium
CSF1	Colony Stimulating Factor 1
CX3CR1	C-X3-C Motif Chemokine Receptor 1
CXCL	Chemokine (C-X-C motif) ligand
DAMPs	Damage-associated molecular pattern molecules
DC	Dendritic cell
DMM	Destabilization of the medial meniscus
DRG	Dorsal root ganglia
ECM	Extracellular matrix
EGF	Epidermal growth factor
FAPα	Fibroblast activation protein – alpha
FLIP	Flice Inhibitory Protein
GM-CSF	Granulocyte-macrophage colony stimulating factor
HLA-DR	Human Leukocyte Antigen – DR isotype
IFN-γ	Interferon gamma
IgG	Immunoglobulin G
IL-	Interleukin
iNOS	Inducible nitric oxide synthase
JAK	Janus kinase
LTB4	Leukotriene B4
MARCO	Macrophage receptor with collagenous structure
M-CSF	Macrophage colony stimulating factor
MIA	Monoiodacetate
MMPs	Matrix metalloproteinase
NF-κB	Nuclear factor kappa-light-chain-enhancer of activated B cells
NGF	Nerve growth factor
NKA	Neurokinin A
NSAIDS	Nonsteroidal anti-inflammatory drugs
OA	Osteoarthritis
P2X7	P2X purinoceptor 7
PAMPs	Pathogen-associated molecular patterns
PDCD	Programmed cell death protein 1
PPOL	Periprosthetic osteolysis
PTEN	Phosphatase and tensin homolog
RA	Rheumatoid arthritis
RAGE	Receptor for advanced glycation end-products
RANKL	Receptor activator of nuclear factor kappa-β ligand
RASFs	Rheumatoid arthritis synovial fibroblasts
RF	Rheumatoid Factor
SCID	Severe combined immunodeficiency
SEN-P1	Sentrin-specific protease 1
sFASL	soluble Fas Ligand
Siglec 10	Sialic Acid Binding Ig Like Lectin 10
sTNFR2	Soluble tumor necrosis factor receptor 2
SUMO-1	Small ubiquitin-like modifier-1
TFH	T follicular helper cells
TGF	Tumor growth factor
TH	Tyrosine hydroxylase
TIM-3	T cell immunoglobulin and mucin-domain containing-3
TLR	Toll-like receptor
TNFSF14	Tumor necrosis factor superfamily member 14
TNF-α	Tumor necrosis factor alpha
TPH	T peripheral helper cells
TRAP	Tartrate-resistant acid phosphatase
TREM1	Triggering receptor expressed on myeloid cells 1
TrKA	Tropomyosin receptor kinase A
TRPV1	Transient receptor potential cation channel V member 1
VCAM-1	Vascular cell adhesion molecule-1
VMAT-2	Vesicular monoamine transporter 2
